# Neurocraft: software for microscale brain network dynamics

**DOI:** 10.1038/s41598-021-99195-y

**Published:** 2021-10-20

**Authors:** Dimitris Fotis Sakellariou, Angeliki Vakrinou, Michalis Koutroumanidis, Mark Phillip Richardson

**Affiliations:** 1 Real World Solutions, IQVIA, London, N1 9JY UK; 2 Department of Basic and Clinical Neuroscience, Institute of Psychiatry, Psychology and Neuroscience, King’s College London, London, UK; 3 Department of Neurophysiology and Epilepsy, St Thomas’ Hospital NHS Trust, London, UK

**Keywords:** Dystonia, Encephalopathy, Epilepsy, Computational science, Computer science, Statistics, Software

## Abstract

The brain operates at millisecond timescales but despite of that, the study of its functional networks is approached with time invariant methods. Equally, for a variety of brain conditions treatment is delivered with fixed temporal protocols unable to monitor and follow the rapid progression and therefore the cycles of a disease. To facilitate the understanding of brain network dynamics we developed Neurocraft, a user friendly software suite. Neurocraft features a highly novel signal processing engine fit for tracking evolving network states with superior time and frequency resolution. A variety of analytics like dynamic connectivity maps, force-directed representations and propagation models, allow for the highly selective investigation of transient pathophysiological dynamics. In addition, machine-learning tools enable the unsupervised investigation and selection of key network features at individual and group-levels. For proof of concept, we compared six seizure-free and non seizure-free focal epilepsy patients after resective surgery using Neurocraft. The network features were calculated using 50 intracranial electrodes on average during at least 120 epileptiform discharges lasting less than one second, per patient. Powerful network differences were detected in the pre-operative data of the two patient groups (effect size = 1.27), suggesting the predictive value of dynamic network features. More than one million patients are treated with cardiac and neuro modulation devices that are unable to track the hourly or daily changes in a subject’s disease. Decoding the dynamics of transition from normal to abnormal states may be crucial in the understanding, tracking and treatment of neurological conditions. Neurocraft provides a user-friendly platform for the research of microscale brain dynamics and a stepping stone for the personalised device-based adaptive neuromodulation in real-time.

## Introduction

The brain switches very rapidly between different brain states, characterised by specific activity in networks of neurons and brain regions, many times per second. Some diseases and conditions affecting the brain involve rapid dynamic switching between normal and abnormal network states which may be characterised by short-lived electrophysiological features. For example, in epilepsy, a key role in the formation and evolution of abnormal states and their pathogenic networks is thought to be played by Interictal Epileptiform Discharge (IED)^1^. IEDs are microscale EEG elements, typically consisting of single or multiple cycles of spikes or spikes-and-waves, lasting from a few milliseconds to a couple of seconds. Despite that numerous neurophysiological events in the temporal microscale have been widely documented and linked to a variety of abnormal states and neurological conditions^2–7^, their dynamic network features remain largely unexplored.

There is much evidence to suggest that the common mechanistic principle across the numerous causes of epilepsy is either the abnormal neuronal population dynamics, or the abnormal connectivity between neuronal populations or both^8^. This evidence strongly suggests that epilepsy is a disease of abnormal network organization of brain areas and the connections between them^9^. Typically, analyses of abnormal states are derived from long EEG-epochs (seconds-minutes) that include abnormal but also normal brain activity. Therefore, any “brain state” existing in the temporal microscale is likely to be hidden amongst many other brain states and not optimally represented by a multi-second average.

Multiple academic software packages exist for the calculation of functional connectivity from electrophysiological signals, of which EEGLAB^10^, Fieldtrip^11^, Brainstorm^12^ and MNE^13^ are the most widely used. However, only some of these packages allow for the time-locked temporal and spectral decomposition of connectivity estimates. Furthermore despite the fact that network theory is a mainstay in EEG analysis^14–16^, only a couple of toolboxes^17^ exist specifically for the investigation of graph theoretical measures. Some existing challenges in the estimation of EEG networks are (a) analysis for targeted time–frequency windows (b) temporal evolution of networks (c) estimation of centrality measures that characterise network under investigation and (d) propagation models for the approximation of zones related to investigated EEG event.

As a first step towards addressing the above challenges, Neurocraft aims to provide a user-friendly platform for exploratory data analysis and hypothesis construction beyond descriptive statistics. In order to achieve this, and end to end user interface is supported for the (a) the estimation of connectivities that characterise very brief brain states with ultra-high time and frequency resolution combined with seamless integration for (b) the complex dynamic network investigation tailored for electrophysiological brain signals. In this manuscript we describe a novel method that is featured by Neurocraft and which was developed for the selective estimation of time–frequency connectivity with ultra-high resolution. Furthermore, we describe the comprehensive set of tools Neurocraft features for the metanalysis and manipulation of the complex dynamic network data. These network tools include influence metrics, dynamic centrality investigation, network characterisation and propagation mapping amongst others.

For proof of concept, we investigated network differences between Focal Epilepsy (FE) patients with good and poor outcome from resective surgery (N = 6). We describe in the results section powerful differences in Global Microscale Connectivity (GMC) that were found between the FE patient groups and which required methods available in Neurocraft for their demonstration.

## Methods and results

### Basic functions

**Figure 1:**
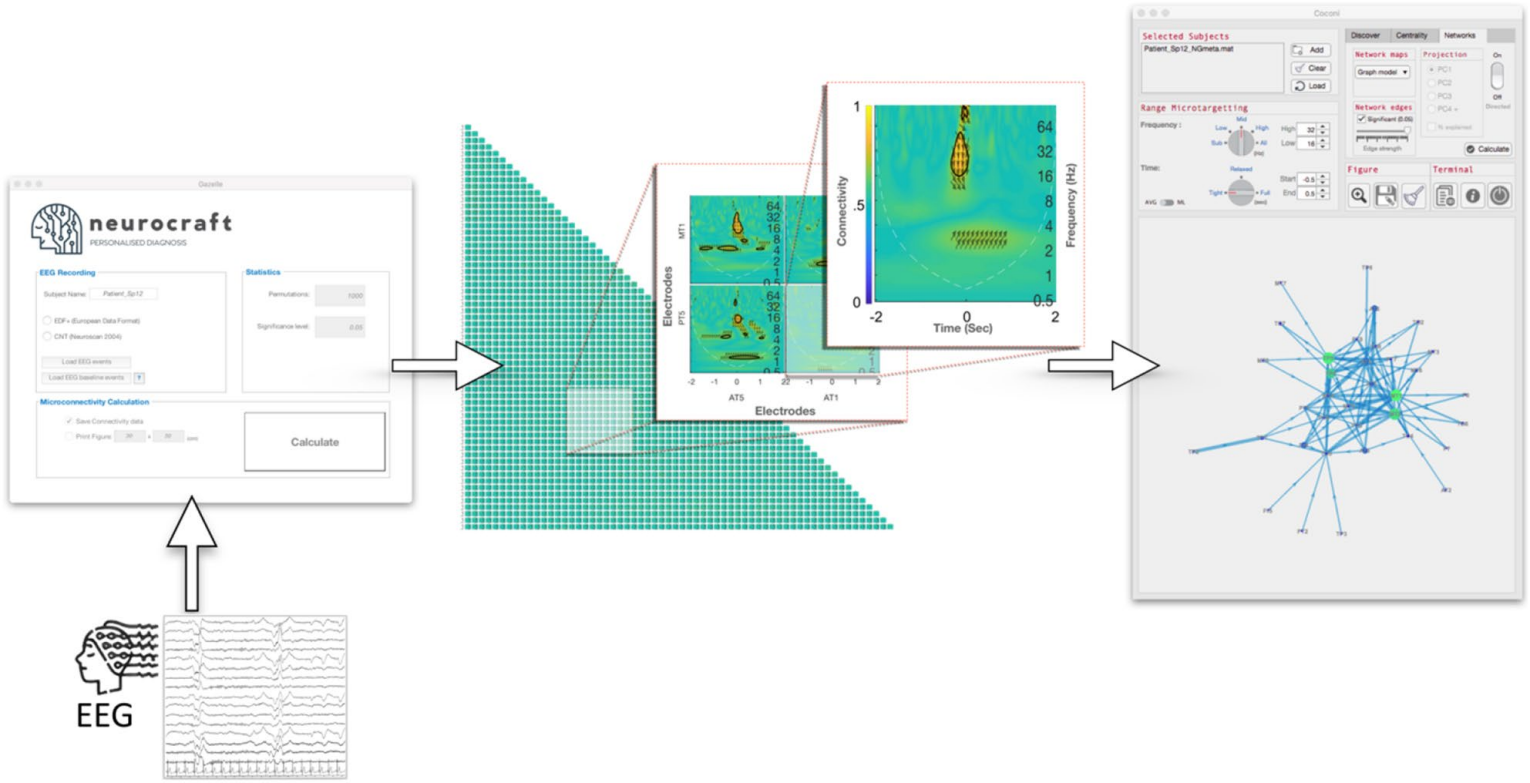
Neurocraft end to end user interface and analysis pipeline (neurocraft 0.1.0, https://www.neurocraft.co.uk/#download).

Neurocraft features a novel connectivity method for the network characterization of electrophysiological datasets at ultra-high time–frequency resolution. Furthermore, this connectivity method is mapped onto robust methodology from graph theory and machine learning and packaged in a comprehensive user-friendly graphical user interface (Fig. 1).

#### Data pre-processing

Neurocraft allows reading of data, event information and channel location from European standard EDF + and Neuroscan formats. Standard data processing functions include selection of channels for analysis, resampling, baseline removal and extraction of data epochs time-locked to annotated events of interest.

### Short-lived connectivity dynamics

In principle, neurobiological phenomena occur at millisecond timescales^18^. The investigation of such short-lived microscale events is the primary focus of Neurocraft, which facilitates the event-selective examination of microscale networks dynamics. To enable the estimation of dynamically varying connectivities between EEG signals here we developed an original method to the authors best knowledge, that.

- Enables optimal time–frequency trade-off for data driven investigations, using continuous wavelet transform.
- Is only sensitive to meaningful synchronisations, by deploying the imaginary part of wavelet transform coherency.
- Features a robust framework for protection against noise, edge effect and random synchronisation by making use of ensemble techniques and bootstrap statistics.

#### Continuous wavelet transform

In principle, neurophysiological signals are non-stationary processes whose statistical properties change with time. The dynamic spectral properties of non-stationary processes can traditionally be estimated using either the Short-Time Fourier Transform (STFT) which is a windowed Fourier transform trading off resolution in frequency to achieve resolution in time, or Continuous Wavelet Transform (CWT) which is a function that is localised in both frequency and time. Generally, CWT is considered a means for optimal time–frequency analysis (TFA) of non-stationary signals when the investigation is exploratory and not hypothesis driven^19^ (Appendix A).

#### Wavelet transform coherency

The time–frequency relationship between two non-stationary processes can be detected by the use of time–frequency coherency, which can either be based on STFT or CWT. Nonetheless, the CWT based coherency appears to be advantageous due to CWT’s compact support and the optimal trade-off between frequency and time in its resulting Heisenberg boxes^20^. Specifically, localised smoothing allows the Wavelet Transform Coherency (WTC) to be estimated even for a single trial in contrast with the STFT Coherency (STFC), which needs to be estimated by averaging across trials to enable localisation in both time and frequency^21^. Importantly, WTC exhibits better frequency and time resolution compared to STFC^22^, at the cost of higher processing load.

To estimate time–frequency coupling interactions with state of the art resolution, here we make use of WTC (Appendix B).

In principal, coherency is strongly influenced by linearity in phase^20^ and non-linear fluctuations in power. These properties of coherency allow for the quantification of dependencies between two time series with regards to their simultaneous values and also to their leading, lagged and smoothed relationships. The sensitivity of coherency and consequently WTC to phase, requires the mother wavelet used for its calculation to be complex.

The Morlet wavelet^23^ is a complex sine wave within a Gaussian envelope and is defined as

1 $$\psi\left(\tau\right) = \pi{^{{{\frac{1}{4}}}}}e^{i\omega_{0}\tau} e^{{{\frac{{-{\tau^{2}}}}{2}}}}$$

where ${\omega }_{0}=2\pi {\text{f}}_{0}$ is the non-dimensional frequency. The Morlet wavelet has been widely used in EEG TFA^24^ and offers an intuitive bridge between frequency and time information. Here, the calculation of WTC computation (Appendix B) uses the analytic Morlet wavelet. The spread of the wavelet’s energy in time and frequency determines the minimum and maximum scales^25^, here taken to be${\omega }_{0}=6$to satisfy the admissibility condition^26^^26^. The valid range of number of octaves is between 1 and $\left\lfloor {{\text{log}}_{{2}} ({\text{f}}_{{\text{s}}} \Delta {\text{t}})} \right\rfloor - 1$ where ${\text{f}}_{\text{s}}$ and Δt the sampling frequency and duration of a x(t) signal. Moreover, neurocraft uses 12 voices per octave and an equal number of scales to smooth. The scales are discretized using the specified number of voices per octave.

#### Ensemble wavelet transform coherency

The EEG signal to noise ratio can often be too low to reliably analyse single events. Many EEG studies use averaging across epochs of realisations of the same event or stimuli to statistically enhance results by implicitly assuming that noise is a zero-mean random variable independent of repetition.

For experimental designs that are event-related (e.g. time-locked repetitions of a trial or investigation of occurrences of a specific neurophysiological phenomenon), neurocraft automatically switches to an ensemble calculation of WTC i.e. estimated WTC, by taking into account n epochs of the same random process (Eq. 2).

2 $$\hat{R}_{{ij}} \left( {\sigma ,\tau } \right) = \frac{{\mathop \sum \nolimits_{{i = 1}}^{n} W_{{ij}} \left( {\sigma ,\tau } \right)}}{{\left( {\mathop \sum \nolimits_{{n = 1}}^{N} S\left( {\left| {w_{i} \left( {\sigma ,\tau } \right)} \right|^{2} } \right) \cdot \mathop \sum \nolimits_{{n = 1}}^{N} S\left( {\left| {w_{j} \left( {\sigma ,\tau } \right)} \right|^{2} } \right)} \right)^{{1/2}} }}$$

where $\text{n}=1,\dots ,\text{N}$ the number of event epochs. In principle, when a number of repetitive realisations of the same random process is present it is sufficient to rely on ergodicity, with stationarity not being anymore a prerequisite to estimate coherency ^27,28^.

#### The imaginary part wavelet transform coherency

In EEG, spurious connectivity measurements can occur due to the “volume conduction” effect, where a single source of activity can contribute to measurements in adjacent electrodes. This spread of activity despite being measurable by multiple sources will principally have a zero shift in inter-area synchrony measurements, as a signal cannot be time-lagged to itself. Other noise sources can evade EEG recordings in the form of inter-area zero-phase activations.

For this reason, and in order to interpret coherency as a measure that reflects neuronal interaction between areas, we use here the Imaginary part of WTC (IWTC)^29^.

3 $$\text{IWTC}=\text{Imag}\left({\widehat{\text{R}}}_{\text{ij}}\left(\upsigma ,\uptau \right)\right)$$

##### Cone of influence

**Figure 2:**
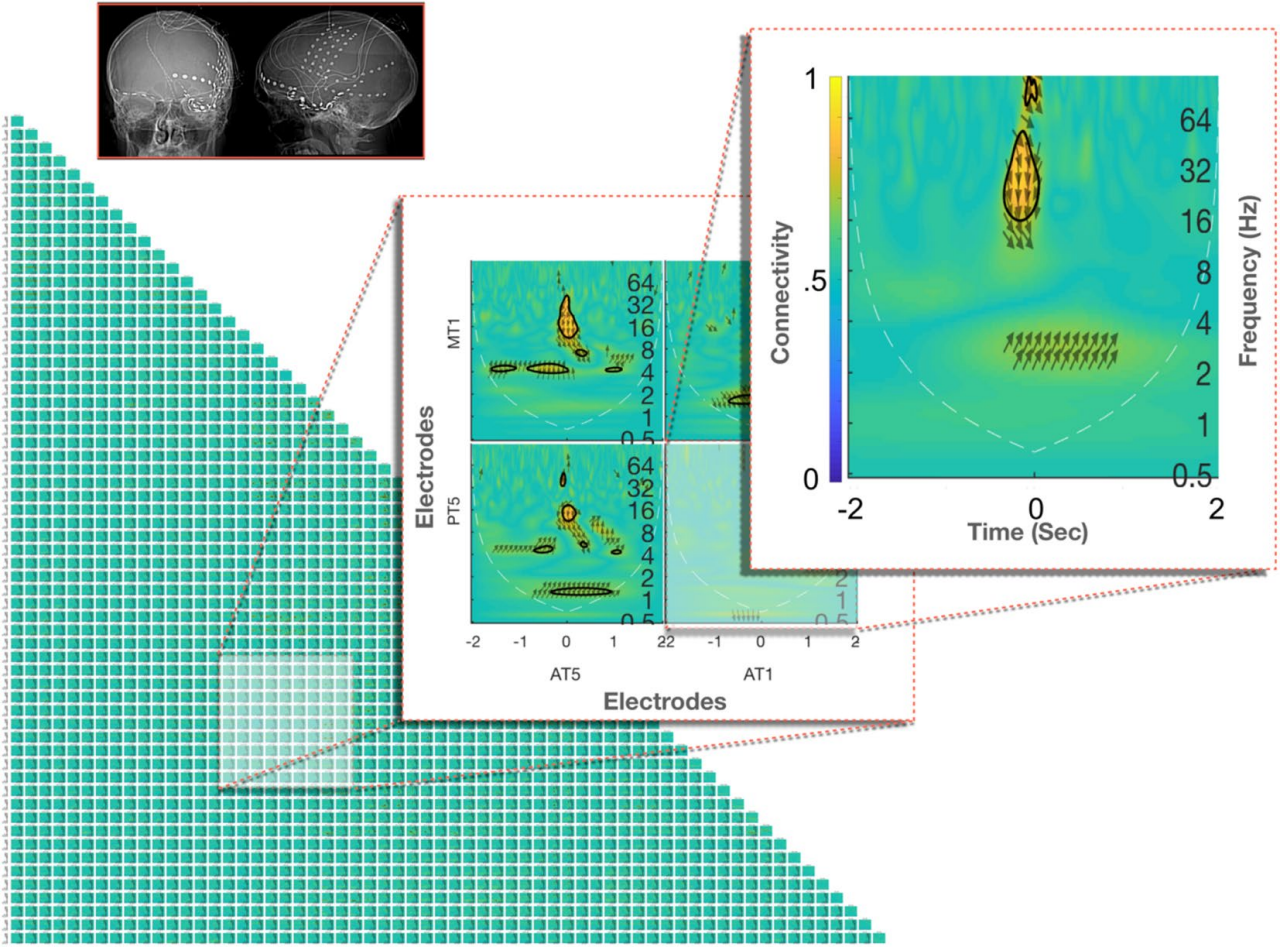
Subject-level connectivity map. Areas in X–Y axes and connection strength between x–y pairs of electrodes indicated in warm and cold colours. Up and down-ward direction of arrows indicative of information flow from the electrodes on the X axis towards the ones in Y and vice versa. The connectivity for a pair of electrodes is estimated over the time and frequency domains (x and y axis of subgraphs) allowing for the characterisation of micro-scale network dynamics around and EEG event (t = 0 s).

The CWT estimate at lower frequencies may suffer from edge effects, as the broader wavelets extend to areas outside the data window^30^ (Zhan et al., 2006b). This phenomenon is referred to as the cone of influence (COI)^20^, it is equal to half of the wavelet length at each scale. Neurocraft automatically plots the COI with a dashed line in the time–frequency connectivity graphs i.e. connectivity maps (Fig. 2).

##### Significance testing against random coherency

Neurocraft makes use of WTC to measure when two processes exhibit stable phase relation on a certain scale for a certain time interval. However, processes in natural systems often exhibit power in a wide range of scales with similar phase evolution patterns. Consequently, while two processes may be mathematically coherent by exhibiting consistency in phase they are not necessarily coupled for real-world reasons.

Additional to ensemble WTC, to statistically identify intrinsic coupling between brain areas we apply non-parametric bootstrapping by constructing surrogate data using randomly selected background EEG epochs under the null hypothesis of independence^31^. Subsequently, connectivity values exceeding a certain quantile of the surrogate distribution signify intrinsic properties^32^. This methodology is (a) flexible in the selection of background EEG epochs to model and test against random connectivity and therefore (b) minimises assumptions underlying the surrogate model. A variety of experimental EEG setups and systems can hence be supported in minimising influences from systemic systemic artefactual signal synchronisations.

In specific, this is a user-driven approach to test significance against systemic coherency. The EEG epochs for the construction of surrogate data models are selected by the user under the null-hypothesis (i.e. independence) along with the number of bootstrap resamples^32^. In connectivity maps, significant connectivity values are highlighted in black contour bands.

#### Visualisation of microscale connectivity dynamics

At this subject level, the time–frequency connectivity bivariate interactions are visualised via connectivity maps (Fig. 2). In the subplot boxes of each map, the evolution of connectivity in time and frequency for a pair of electrodes is captured in terms of strength and directionality i.e. relative phase relationship. The pair of electrodes relevant to each subplot are denoted in the x and y axes of the master graph. Strength of connectivity for each pair of areas is coded cold (green) and warm (red) colours. Additionally, the relative phase relationship between a pair of areas is shown as arrows; (a) Up and down-wards arrow direction denotes information from the electrode on the X master axis towards the Y and vice versa (b) from right to left direction denoting in-phase to anti-phase synchronisation.

### Microscale networks: meta-analysis of interregional dynamic connectivity

The subject-specific connectivity maps contain a vast amount information over the time, frequency and space domains. To enable the detailed examination of these complex dynamic datasets, neurocraft features a network metanalysis module.

Comprehensive network metanalysis is enabled by user-based time–frequency range targeting, subject and group level analysis, node influence and centrality metrics, network simulations and propagation models as well as dynamic network analysis.

#### Time–frequency targeting

As network, we define here a static representation of the dynamic connectivity values within a time–frequency window selected by the user. Within this targeted window, the set of discrete EEG areas that exhibit significant synchronicity^33^ and are causal with each other are defined as nodes of the static network or otherwise snapshot.

We define a network snapshot as

4 $${\text{G}}_{\text{t},\text{f}}^{\mathfrak{D}}=(\mathcal{V},{\text{E}}_{\text{t},\text{f}} )$$

during a finite time epoch [${\text{t}}_{\text{start}}$, ${\text{t}}_{\text{end}}$] and frequency range [${\text{f}}_{\text{low}}$, ${\text{f}}_{\text{high}}$] that consists of $\mathcal{V}$ vertices and time–frequency edges ${(\text{u},\upnu )}_{\text{i},\text{j}}^{\text{k},\text{l}}\in {\text{E}}_{\text{t},\text{f}}$ that exist between u and ν in a time interval [$\text{i},\text{j}$] such that $\text{i}\le {\text{t}}_{\text{end}}$ and $\text{j}\ge {\text{t}}_{\text{start}}$ and in a frequency range [$\text{k},\text{l}$] such that $\text{k}\le {\text{f}}_{\text{high}}$ and $\text{l}\ge {\text{f}}_{\text{low}}$. The network snapshot is calculated by averaging connectivity values of all existing edges within the targeted time–frequency window.

#### Subject and group level analysis

Apart from subject-specific analysis, multiple connectivity datasets can be introduced into neurocraft for group network metanalysis. For the calculation of measures across multiple datasets, grand average is used as a default. Specifically, the grand average is calculated as the mean of connectivity values across subjects within the user defined time–frequency window for each inter-area comparison.

##### Cross-subject network investigation

Despite being commonly used, grand-averaging across subjects may distort results and fail to account for the internal group variability of connectivity results.

Additional to grand averaging, traditional pattern recognition methodology can optionally be used to identify network modules. Neurocraft makes use of Principal Components Analysis (PCA) to identify important subnetworks in relation to the group data variance, as previously demonstrated^16^. Specifically, the first three components of PCA are calculated along with the group variance that each component accounts for. PCA is applied to ${\text{G}}\in {\text{R}}^{{\text{n}}\times {\text{m}}}$, where n rows correspond to subjects and m columns to the connectivity values of each possible pair of electrodes after subject-specific mean normalisation calculated according to the methodology described above. Generally, PCA is calculated as the orthogonal linear transformation of the original, possibly correlated variables into a set of linearly uncorrelated variables i.e. principal components (PC)^34^. Here we use the singular value decomposition for the calculation of PCs. To identify the pattern of connections i.e. network module each PC is associated with, the coefficients of the PCs are down-projected onto the network variables. Importantly, each network module accounts for a percentage of the overall variance of the group of datasets.

#### Centrality measures and nodal influence

Many systems in nature are made by a large number of highly interconnected dynamical units^35^. In such systems, certain nodes have a special role and can be seen as central with respect to a given role and a variety of centrality measures have been heuristically developed. Centrality measures map to specific roles and can be used to quantify node importance within the network under investigation.

Here, we make available 10 well-established centrality measures for detecting important nodes in weighted non-directed and directed graphs. Non-directed measures include weighted degree, closeness and eigenvector centralities. Directed centrality measures include weighted indegree, outdegree, incloseness, outcloseness, betweenness, hubs and pagerank centralities. The formulas for each measure are given in Appendix A.

##### Characteristic centrality

The importance of detecting influential nodes in complex systems has brought a wide-scale adoption of network theory in diverse scientific disciplines. However, successful means for node detection may vary between network systems depending on their intrinsic attributes such as topology, directionality, partitioning or connection weight. Therefore, the selection of a centrality measure to appropriately rank nodes according to their importance can be challenging considering the great variety of benchmark influence metrics.

Neurocraft makes use of traditional dimensionality reduction methodology to address the above. In specific, we provide a statistical framework for comparing and prioritising centrality measures based on contribution criteria. Specifically, centrality measures are being considered as variables for the calculation of a PCA. PCA takes place across all available connections in the network. In this way, we estimate which centrality measures are correlated with principal components and therefore hold the most information for the connections of the investigated network. Effectively, we denote centrality features as of key importance in relation to their respective contribution to the principal component that account for the most of the variance in the network dataset. The sorted contributions of variables i.e. measures of centrality are visualised and signify the ones that account for the bigger part of the data variance.

The above process is a data-driven approach for choosing a centrality measure according to which influential nodes can be appropriately detected, in terms of contribution criteria. It is important to note that each centrality measure is linked with specific node attributes of the network under investigation. Therefore, characteristic centrality can also serve as a descriptive measure for the network as a whole.

##### Network models and visualisation

A variety of topographical and nodal visualisations are available through neurocraft in order to observe, manipulate and analyse network snapshots:

- Heatmaps: Rows and columns represent nodes to reflect node to node connectivity levels in colour, for all available nodal pairs.
- Graph model: Pictorial representation of the nodes and edges for the selected network snapshot. The topographical positioning of nodes is determined according to a force-directed layout to reflect inherent symmetry and centrality features of the system^36^. Centrality values are expressed in nodal size and colour, for the selected centrality measure. Connectivity levels between a pair of areas are expressed in edge thickness. Additionally, optional trimming of non-significant edges is available, along with manual edge thresholding for weaker connections. For directed graphs, directionality for a pair of connections is represented in arrows.
- Dendrogram model: For directed networks, a hierarchical network representation in which the nodes are drawn in horizontal directionality layers with the edges generally directed downwards^37^. In its ideal form, this model would depict the propagation patterns for the transmitted information across the directed network, in which all edges maintain a consistent direction and no pairs of edges cross. However, cycles are expected to exist in graphs and especially in those representing dynamic natural systems. Layered graph layout systems attempt to minimise the number of edges that cross along with inconsistent directionalities. Nonetheless, this problem is NP-hard^38^ and therefore this depiction is highly experimental and should be interpreted always in context of the classical heatmap and force-directed models.

##### Dynamic network and centrality modelling

While microtargeting offers a means to estimate network “snapshots”, these representations are built by collecting information over a period of time and are static despite their microscale attributes. However, brain dynamics switch very rapidly between different brain states many times per second^18^. A dynamic representation of the network structure aggregates information from multiple snapshots over time and in complex brain networks such representations can leverage not only structural/spatial features but also their temporal progression. To enable time-resolved investigation of network properties, we introduce the dynamic centrality feature.

We define as a dynamic network observation as:

5 $${\text{G}}_{0,\text{T}}^{\mathfrak{D}}=(\mathcal{V},{\text{E}}_{0,\text{T}} )$$

during a finite time epoch [0, T], where ${\text{t}}_{\text{start}}=0$ and ${\text{t}}_{\text{end}}=\text{T}$ without loss of generality, that consists of $\mathcal{V}$ vertices and temporal edges ${(\text{u},\upnu )}_{\text{i},\text{j}}\in {\text{E}}_{0,\text{T}}$ that exist between u and ν in a time interval [i,j] such that ${\text{i}}\le {\text{T}}$ and $\text{j}\ge 0$. Essentially, we define here as a dynamic network representation a set of $\mathcal{V}$ vertices with a set of edges that change over time.

This discretisation of temporal dynamics into a sequence of network snapshots is necessary to apply graph theoretical analysis in a dynamic manner and has been adopted in a variety of network theory fields in the past^39^. The time period is divided in fixed discrete steps $\{1,\dots ,\text{n}\}$ with ${\text{w}}={\rm T}/{\rm n}$ denoting the window size and ${\text{G}}_{\text{t}}=\{{\text{G}}_{1},\dots ,{\text{G}}_{\text{n}}\}$ with $1\le {\text{t}}\le {\text{n}}$ the aggregate graph consisting of $\mathcal{V}$ vertices and temporal edges $(\text{u},\upnu )\in {\text{E}}_{\text{t}}$ that exist between u and ν in a time interval [i,j] such that $\text{i}\le {\text{w}}\cdot {\text{t}}$ and $\text{j}>\text{w}\cdot (\text{t}-1)$. Essentially, ${\text{G}}_{\text{t}}$ is the tth temporal snapshot of ${\text{G}}_{0,\text{T}}^{\mathfrak{D}}$ at the tth time window. Consequently, centrality is calculated across all ${\text{G}}_{\text{t}}$ snapshots and dynamic centrality is presented as either a multi-nodal time resolved graph or as a time resolved centrality mean over all nodes. All of the available measures can be used for the dynamic centrality estimation. The w window size can be defined by the user, for a flexible time resolution versus computational cost trade-off.

## Results

### In silico demo of WTC and IWTC

We demonstrate the usability of WTC and its imaginary part in synthesised signals. WTC and IWTC is tested for a pair of non-stationary time series defined as

$$x = \left\{ {\begin{array}{*{20}c} {\cos \left( {2\pi \cdot 10t} \right) + \psi ,~~t \ge 0.5~and~t < 1.1} \\ {\cos \left( {2\pi \cdot 45t} \right) + \psi ,~~t \ge 0.2~and~t < 1.4} \\ \end{array} } \right.$$

and

$$\text{y}=\left\{\begin{array}{c}\text{sin}\left(2\uppi \cdot 10\text{t}\right)+\psi , \quad t\ge 0.7 and t<1.2\\ sin\left(2\uppi \cdot 45\text{t}\right)+\psi , t\ge 0.5 and t<1.6\end{array}\right.$$

with $\uppsi$ a white noise process of random uncorrelated variables, at 0.05 scales.

**Figure 3:**
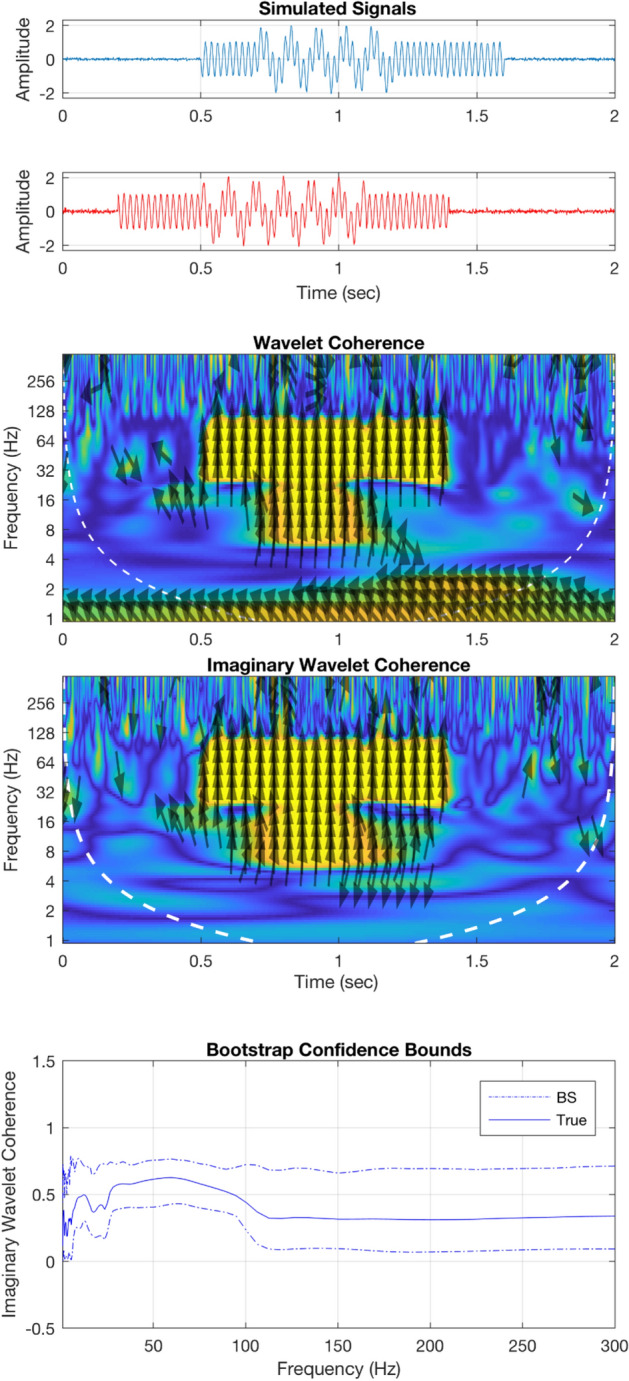
Wavelet coherence simulations. (Top) In blue and red, synthesised non-stationary signals. (Middle) WTC and IWTC graphs depict the co-occurrences of $45\text{Hz}$ and $10\text{Hz}$ rhythms in the pair of signals. Arrows depict the phase shift between a pair of signals, here vertical with upward direction suggesting a shift of $\varphi = {\pi \mathord{\left/ {\vphantom {\pi 2}} \right. \kern-\nulldelimiterspace} 2}$ flow of information from the y (red) towards the x (blue) signal. (Bottom) True WTC and estimated 95% confidence bounds with the bootstrap approach (dot dashed).

In Fig. 3, the co-occurrence of similar oscillatory activity (fast $45\text{Hz}$ and slow $10\text{Hz}$) in the pair of signals are accurately captured by the WTC and IWTC, as presented in the relevant graphs. Additionally, the onset/offset of sync activations is accurate with millisecond precision. In terms of phase, the vertical arrows accurately depict the $\varphi = {\pi \mathord{\left/ {\vphantom {\pi 2}} \right. \kern-\nulldelimiterspace} 2}$ shift between x and y. The upward arrow direction correctly suggests a directional relationship for the pair from y (red) to x (blue), since fast and slow rhythms are initially present in y and later become apparent in x. Random sync activations irrelevant to the content of the pair of signals, can safely be attributed to the ψ white noise processes. Such activations appear to be less apparent in IWTC, which is generally less prone to type I errors.

### Focal epilepsy: real world data

We sought to test the applicability of our tools in six exemplar Mesial Temporal Lobe Epilepsy (MTLE) patients who had undergone epilepsy surgery at King’s College Hospital, London, UK. In groups of patients with good (Engel I: free of disabling seizures; Engel II: rare disabling seizures (“almost seizure-free”)) and poor (Engel III: worthwhile improvement; Engel IV: no worthwhile improvement) post-operative outcome, we examined the functional connectivity features (Table 1).

**Table 1:** Details of patients with Mesial Temporal Lobe Epilepsy.

	Gender	Age	MRI diagnosis	Num of Channels	Recording duration (days)	Num of seizures	Engel Simple
**Good outcome**							
1	Male	32	Normal-unspecific	63	9	8	Favourable (I-II)
2	Male	25	Normal-unspecific	24	3	5	Favourable (I-II)
3	Male	18	Normal-unspecific	99	11	7	Favourable (I-II)
**Bad outcome**							
4	Male	20	Normal-unspecific	62	6	3	Not favourable (III-IV)
5	Male	50	Normal-unspecific	60	16	5	Not favourable (III-IV)
6	Female	27	Normal-unspecific	60	14	3	Not favourable (III-IV)

Num of seizures: number of seizures during intracranial EEG investigation as identified by visual inspection.

ECoG was used in pre-surgical evaluation, and here we define a functional connectivity network based upon ECoG recordings. On average, 62 intracranial temporal and frontal electrodes were available, covering successfully the epilepsy-related areas as identified and targeted by the clinicians. Preoperative ECoG lasted ten days on average. In the preoperative data, we identified per patient at least 120 Interictal Epileptiform Discharge (IED), a hallmark graphoelement of epileptic EEG. IEDs are thought to play a key role in the formation and evolution of pathogenic networks in epilepsy^40^.

All procedures were carried out in accordance with guidelines and protocols approved by the Ethical Committee of King’s College Hospital (reference number 99–017), including acquisition of informed consent for all subjects of the study. The data can be accessed upon request from the authors, according per the Ethical Committee agreement.

#### IED network differences in focal epilepsy

**Figure 4:**
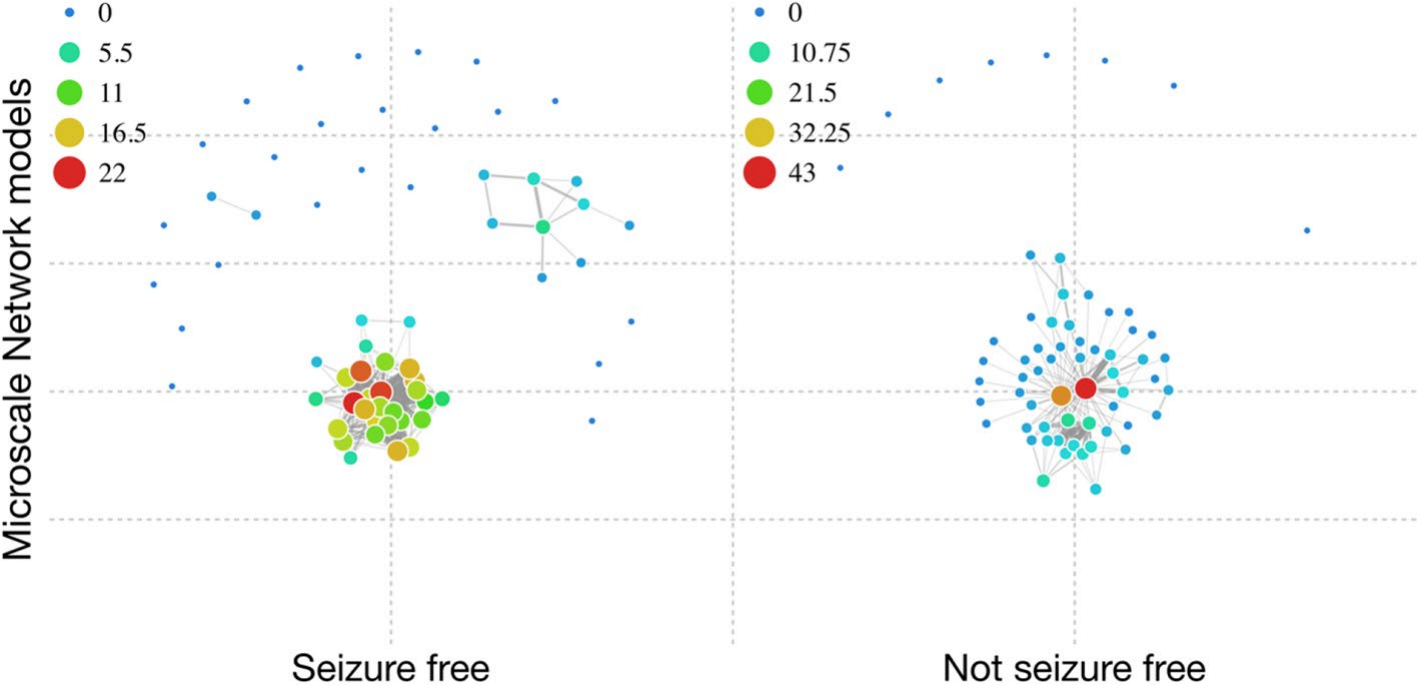
Comparison MTLE patients in groups of positive and negative resective surgery outcome. The presented force-directed simulations were calculated in preoperative recordings and predicted postoperative outcome. The networks activated during IEDs are much more strongly coupled in the negative outcome group, sustaining more connections and widespread structure.

Powerful network organisation differences were discovered between good and poor outcome MTLE patient groups. Specifically in the preoperative intracranial ECoG recordings of each subject, a 2 s period around IED events [− 2,2]sec was selected (Neurocraft > microtargeting > Time > ”Full”) with a frequency window of [0, 122]Hz (Neurocraft > microtargeting > frequency > ”Full”) and the respective the Global Microscale Connectivity (GMC) was calculated (Neurocraft > levels tab > Global Connectivity Levels > ”calculate”). GMC levels related to IEDs were found to be significantly different between groups, with an effect size of 1.271 (Fig. 4). The significant differences in GMC, signifies that the microscale networks associated with short epoch surrounding IEDs are much more strongly coupled in the group that did not have a good outcome from surgery, suggesting that strong coupling was retained even after resection. Crucially, the network features in the MTLE groups were calculated in preoperative data and predicted postoperative outcome.

## Discussion

There is growing evidence that the common mechanistic principle across many neurological disorders (such as epilepsy and movement disorders) is disruption to normal neuronal network dynamics. Here, we propose a novel method for decoding neuronal network dynamics in terms of temporal variation, frequency band and location can potentially reveal network markers for disease detection. Additionally, a variety of exploratory network analysis pipelines are proposed for the first time to our knowledge. These methodologies are packaged in a user friendly suite with a standalone MATLAB UI: neurocraft. This end-to-end network dynamics tool features flexible modules for importing data, significance testing and visualising network results. We demonstrate the usability of neurocraft in an exemplar epilepsy dataset where differences are identified between the networks of good and bad surgery outcome patients.

### Continuous wavelet coherency

The widely adopted Short Time Fourier Transform (STFT) performs “uniform tiling” over the time–frequency domain which enables expert frequency resolution for a user-specified band. In this way STFT is well suited for the analyses of signals whose frequency is known a-priori. However, STFT is less than ideal for investigations where certain frequency activations are not known and remain yet to be discovered. As opposed to STFT, CWT features a “wavelet dyadic tiling” which segments the time–frequency domain flexibly providing a better time–frequency trade-off. In this way, CWT and consequently its coherency measures allow tracking of activities at the entirety of the frequency range providing a better platform for the data-driven investigation of brain networks.

### Significance testing against random coherency

When the underlying mechanism of a process is well understood, significance can be tested against realisations generated by simulation models. While this parametric methodology is for many scenarios well suited e.g. geophysical time series and Monte Carlo methods against red noise, it may not as appropriate in the context of EEG. EEG signals vary wildly between systems and setups, subjects, their cognitive state and the pathophysiology that may underlie them. For this reason instead of attempting to simulate the EEG processes, a non-parametric framework is proposed here that uses the data distribution of each study to test against random connectivity. This model-agnostic approach enables significance testing for a variety of EEG setups and recordings that may vary from the routine EEG experiment.

### Dimensionality reduction

A collection of issues arise together with the increase of the feature space, making the analysis of high-dimensional datasets challenging. Essentially in such datasets, the average and minimum distance between datapoints is increased because of this “dilation” across the high number of dimensions. This sparsity in the data makes datapoints to appear distant and dissimilar making the detection with similar properties among them challenging. The EEG network space often is high-dimensional and to tackle relevant issues neurocraft employs PCA to detect patterns that potentially characterise the majority of a group of subjects under investigation. In this way the plethora of activated network connections are distilled down to their most important projections so that the network patterns are simplified without losing important traits.

### Weaknesses, gaps and future plans

Neurocraft currently consists of two separate UI views, primarily due to limitations from MATLAB. Although data size limits will depend on computational budget, a number of neurocraft methods are not optimised for speed and memory handling. To provide with a more flexible and effective solution in this respect, we aim refactor a significant part of neurocraft so that distributed computing and multithreading is more widely supported. Moreover, the inter-area connectivity matrices are currently stored in MATLAB cells. The process of storing and reading these datasets is not optimised for its memory handling. In the future we aim at improving the memory footprint of these functions to reduce execution times and space usage of the export files, making the overall user experience smoother. Finally, although Quality Assurance (QA) has been thoroughly performed in development, Quality Control (QC) was limited to abide publisher rules. We aim at performing thorough QA/QC as the user base grows after the publication of the platform. Finally, neurocraft uses standard PCA for a variety of tasks however this method is not able to address nonlinear dimensionality reduction tasks and therefore such tools may benefit from methods like kernel PCA or multi-layer autoencoders.

### Potential applications

Outputs from neurocraft could help to inform dynamically-modulated brain therapy. For example, in neuromodulation such as Deep Brain Stimulation for a range of brain conditions, the therapy remains static over weeks/months however patients’ disorders and symptoms are unique and change over minutes/hours. In tackling these issues, adaptive neuromodulation systems attempt to identify pathological signatures and adapt the stimulation output in order to stabilise the pathogenic circuit in a closed loop^41–43^. However, a particular challenge in adaptive systems is what signal should be tracked^44^. Neurocraft’s ultra-high time resolution allows the discovery of “fast” neural signatures reflecting brain network dynamics, allowing to quickly track abnormalities as they take place. This rapid abnormality detection could potentially provide a powerful framework for personalised adaptive neurostimulation treatment in an automated, homeostatic loop.

ERP studies could also benefit from neurocraft tools and pipelines. In many neurological conditions, like Parkinson’s disease^45^, dystonia^46^ and attention deficit hyperactivity disorder^47^ brain functionality is evaluated by means of stereotyped electrophysiological responses to a stimulus. Beyond amplitude and morphology, these neural responses could be further investigated with neurocraft to estimate the network response to a stimuli, the areas that being engaged and how those evolve in the peri-stimuli epoch. Furthermore, neurocraft applications could generalise to other multichannel series data such as EEG-EMG^48^ and can contribute in the analytical characterisation of topological and spectral patterns of synchronisations and how these evolve at short temporal scales.

Beyond the application of these tools as a descriptive tool for clearly defined neurophysiological events, neurocraft provides a platform for exploratory investigations at distant peri-event timepoints; Network behaviour leading up to the onset of an annotated event can be tracked and characterised based on nodal activity or at group-level networks can be investigated for potentially important subnetworks in relation to the group data variance. Beyond descriptive statistics, these pipelines essentially suffice exploratory data analysis and are aimed at hypothesis construction by collecting experimental observations.

## Conclusion

A variety of remarkable EEG tools have been developed in the last decade enabling the large-scale adoption of computational tools by the neuroscience community. Along the lines of these contributions, neurocraft aims to bridge the plethora of steps from EEG clinical records to network analytics and modelling, providing an end-to-end unified platform. Importantly, along a user-friendly interface, neurocraft features a variety of novel methodologies to address important questions that often arise in the study of large real-world brain networks and their dynamics. We hope this is a first step of an open source medium that connects clinical and engineering experts in the study of brain network dynamics and disorders.

## Supplementary Information

Supplementary Information.
