# Supplementary material for: Neurocraft: software for microscale brain network dynamics
Source: Sci Rep. 2021 Oct 20;11:20716. doi: 10.1038/s41598-021-99195-y (PMC8528833; doi:10.1038/s41598-021-99195-y)
Supplement: Supplementary file 1 — Supplementary Information. [file 41598_2021_99195_MOESM1_ESM.docx]

Appendix:

a.

A wavelet transform decomposes a signal into the time frequency space by successively convolving the time series with scaled and translated versions of a wavelet function $\psi_{0}$ (Mallat, 1998). The CWT of a signal $x(t)$ is defined as:

Equation 6

$$w_{x}\left( \sigma,\tau\right)=\int_{-\infty}^{\infty} x\left( t \right)\psi_{a,b}^{⋇}\left( t \right)dt$$

Where

Equation 7

$\psi_{\sigma,\tau}\left( t \right)=\frac{1}{\sqrt{\sigma}}\psi\left( \frac{t-\tau}{\sigma} \right)$

$\psi\left( t \right)$ the mother wavelet, * denotes the complex conjugate, and *σ* and *τ* the scale and translation parameters. The scale parameter can be converted to frequency by satisfying ${f=f}_{0}/\sigma$, where $f_{0}$ a chosen characteristic frequency defined as the bandpass centre of the wavelet energy spectrum (Addison, 2002).

b.

WTC is a correlation measure between two signals based on their wavelet transforms. Given two $x_{i}\left( t \right)$ and $x_{j}\left( t \right)$ processes and their wavelet transforms $w_{i}\left( \sigma,\tau\right)$ and $w_{j}\left( \sigma,\tau\right)$, the wavelet cross-spectrum is defined as

Equation 8

$$W_{ij}\left( \sigma,\tau\right)=S\left( w_{i}^{*}\left( \sigma,\tau\right)w_{j}\left( \sigma,\tau\right) \right)$$

where *S* is the smoothing operator in time $s_{\tau}$ and scale $s_{\sigma}$ which, in order to provide a constant variance for all scales, its averaging kernel operates according to the reproducing kernel [32]. Consequently, the time-frequency wavelet coherency is defined as

Equation 9

$$R_{ij}\left( \sigma,\tau\right)=\frac{W_{ij}\left( \sigma,\tau\right)}{\left( S\left( \left| w_{ij}\left( \sigma,\tau\right) \right|^{2} \right)\cdot S\left( \left| w_{j}\left( \sigma,\tau\right) \right|^{2} \right) \right)^{1/2}}$$

References

Addison, P.S., c2002. The illustrated wavelet transform handbook : introductory theory and applications in science, engineering, medicine and finance /. Institute of Physics Publishing, Bristol

Mallat, S.G., 1998. A wavelet tour of signal processing. Academic Press, San Diego.
